# Unilateral pedicle screws asymmetric tethering: an innovative method to create idiopathic deformity

**DOI:** 10.1186/1749-799X-2-18

**Published:** 2007-10-31

**Authors:** Yonggang Zhang, Yan Wang, Guoquan Zheng, Xuesong Zhang, Ruyi Zhang, Wei Zhang

**Affiliations:** 1 Department of Orthopaedics, General Hospital of Chinese PLA, Beijing 100853, China

## Abstract

**Objective:**

To evaluate the feasibility of the method that unilateral pedicle screws asymmetric tethering in concave side in combination with convex rib resection for creating idiopathic deformity.

**Summary of background data:**

Various methods are performed to create idiopathic deformity. Among these methods, posterior asmmetric tethering of the spine shows satisfying result, but some drawbacks related to the current posterior asymmetric tether were still evident.

**Materials and methods:**

Unilateral pedicle screws asymmetric tethering was performed to 14 female goats (age: 5–8 week-old, weight: 6–8 kg) in concave side in combination with convex rib resection. Dorsoventral and lateral plain radiographs were taken of each thoracic spine in the frontal and sagittal planes right after the surgery and later every 4 weeks.

**Results:**

All animals ambulated freely after surgery. For technical reasons, 2 goats were excluded (one animal died for anesthetic during the surgery, and one animal was lost for instrumental fail due to postoperative infection). Radiography showed that 11 goats exhibited scoliosis with convex toward to the right side, and as the curve increased with time, only 1 goat showed nonprogressive. The initial scoliosis generated in the progressors after the procedures measured 29.0° on average (range 23.0°–38.5°) and increased to 43.0° on average (range 36.0°–58.0°) over 8 to 10 weeks. The average progression of 14.0° was measured. The curvature immediately after tethering surgery (the initial Cobb angle) did have a highly significant correlation with the final curvature (p < 0.001). The progressive goats showed an idiopathic-like deformity not only by radiography, but in general appearance.

**Conclusion:**

Unilateral pedicle screws asymmetric tethering is a practical method to create experimental scoliosis, especially for those who would like to study the correction of this deformity.

## Introduction

Till today, the etiology of the scoliosis is still uncertain. Many theories have been proposed to explain its occurrence, and many attempts have been made to establish a suitable experimental model of scoliosis. People have been interested in the animal models not only suitable to investigate the pathogenesy and the development, but also to study correction of scoliosis.

Various optional methods to create the animal model of scoliosis can be found in the literature. MacEwen [1] divided these methods into those using systemic agents and those using localized surgical procedures on the musculoskeletal or nervous system. The former group included aminonitriles[2], [beta]-aminopropionitrile[3]. Additionally, some mutagenic agents were administered to pregnant animals [4,5] However, a prominent character of those deformities induced by systemic agents is the associate deformities of other organs, which is not similar to the idiopathic scoliosis. Thus, these animal models are not ideal for subsequent studies. In addition, most scholars prone to create experimental scoliosis using localized surgical procedures.

Haas [6] and William Nachlas [7] created experimental scoliosis by resection or compression of the epiphysis cartilaginous pate of the vertebra. Carpintero [8] performed Localized surgical procedures on posterior spine to create experimental scoliosis. While, Thomas S [9], Sevastik J et al [10] and Sevastikoglou JA et al [11] succeeded in the field by rib surgery (elong or shorten the rib), and, to some extent, Barrios C [12], Olsen GA [13], and Joe T [14] also succeeded by interrupting the nervous system or musculature selectively. Additionally, Machida [15-17] and Wang XP [18] et al performed pinealectomy on the chick and bipedal rat. There are many similarities in the development of scoliosis in young chickens after pinealectomy and in children with adolescent idiopathic scoliosis. This method bring an experimental scoliosis model to study the pathogenic mechanism, pathologic mechanism, nature course of this phenomenon, and with the expectation of uncover the etiology of the AIS. However, it is well recognized that there is a large phylogenetic gulf between avian or beast and human.

Among these methods, only a few successes have been achieved in large animal models. Braun JT [19-25], performed a posterior asymmetric rigid or flexible tethering with convex rib resection and concave rib tethering on immature goat. During the tethering period, majority of these goats achieved a progressive, structural, lordoscoliotic curve of significant magnitude convex to the right in the thoracic spine. However, as the author noted, despite the close approximation of idiopathic scoliosis in these animal models, several shortcomings related to the posterior asymmetric tether were evident. There are some risks such as neurological complications during the operation procedures. It is not easy to insert or to remove the tether as well.

As we know, pedicle screw, compared to the hook technology, offers less neurologic problems, and can be implanted or removed easily. Pedicle screw was chosen to take the place of the sublaminar hook as described by Braun JT. The left side rib tethering was cancelled to minimally invasive the tissues surrounding the spine. To reduce the elastic recover strength of the opposite side (right side) of the thoracic skeleton, T7-12 rib resection was needed.

## Materials and methods

This study was performed according to the guidelines of the animal experimental center at General Hospital of Chinese PLA.

Surgery was performed on immature goats who were anesthetized with 3%sodium pentobarbital. The anesthetic dose was about 30–40 milligram per kilogram, and the route of administration was vein injection.

### Operative technique

**Figure 1:**
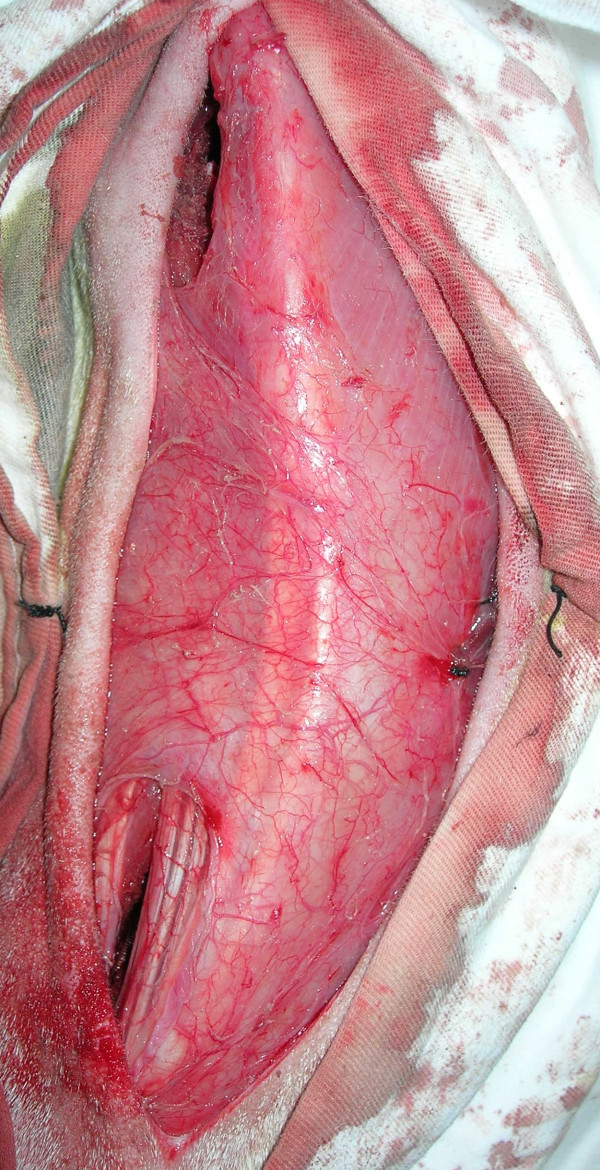
Operative precedure. One posterior paramidline skin incision from approximately T5 to L2 was used to gain access for contralateral cranial and caudal pedicle screws implantation and ipsilateral rib resection.

**Figure 2:**
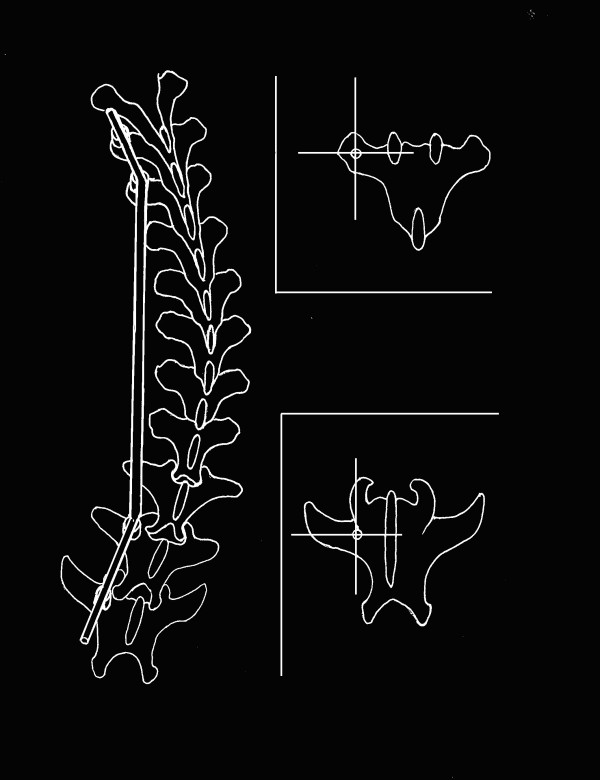
Conception of the internal tether. The superior sketch shows the anchor point of the thoracic vertebra. The inferior sketch shows the anchor point of the lumbar vertebra.

After the anesthetizing procedure and the skin preparation of the operative region, a posterior paramidline skin incision from approximately T5 to L2 was used to gain access for contralateral (left) cranial and caudal pedicle screws implantation and ipsilateral (right) rib resection (Fig 1). Blood vessel forceps was used to dissect the left erector spinae to expose the transverse process for the insertion of two pedicle screws at adjacent levels on left side of the spine, proximally at the T6,7 and distally at L1,2 (Fig 2). The anchor point of the thoracic vertebra was located the intercross point of the midline of the transverse process and the vertical line through the highest point. The anchor point of the lumbar vertebra was located the intercross point of the midline of transverse process and the lateral rim of the superior articular process. The angulations between the direction of T6, 7 pedicle screws and the sagittal plane of spine (angle of crab) were about 30°, while the counterparts of the L1, 2 were about 40°. It was not necessary to dissect amina extensively during this procedure. The pedicle screws served as proximal and distal anchors for the tethering.

Subcutaneous latissimus dorsi was dissected in the right thorax allowed for convex resection of 2 to 3 cm of T7-12 rib. The thirteenth rib, as costa fluctuantes, contributing less to the stability of the spine, was not resected. The rib resection was accomplished in a standard subperiosteal manner without violating the underlying rib bed or pleura.

A prebending stainless steel rod was then passed subcutaneously and submuscularly between the sets of pedicle screws. Firstly, the rod was fixed with proximal two pedicle screws by setscrews, and the spine was subsequently compressed to create a right thoracic scoliosis. The rod was fixed on the distal screws aftermath. Figure 1 shows the two incisions, at the cranial and caudal ends, where the anchor screws were placed and the tether after the screws nut have been tightened.

### Radiographic examination

Dorsoventral and lateral plain radiographs were taken of each thoracic spine in the frontal and sagittal planes right after the surgery and every 4 weeks after the operative procedures. Cobb angles were measured using radiographs. The degrees of coronal and sagittal deformity and vertebral wedging were measured using standard Cobb angle technique.

### Statistical analysis

Statistical analyses were performed using t student test, and the level of statistical significance was set to *P *< 0.05

## Results

All 14 female goats were performed with unilateral pedicle screws asymmetric tethering in concave side in combination with convex rib resection (age: 5–8 week-old, weight: 6–8 kg). All animals ambulated freely after surgery. For technical reasons, 2 goats were excluded. One animal died for anesthetic during the surgery (overdose sodium pentobarbital may slow down the respiratory frequency and the heart rate, the risk may increase when a compressing strength was performed on the thoracic cage), the other animal was lost for instrumental fail contributed by postoperative infection.

**Figure 3:**
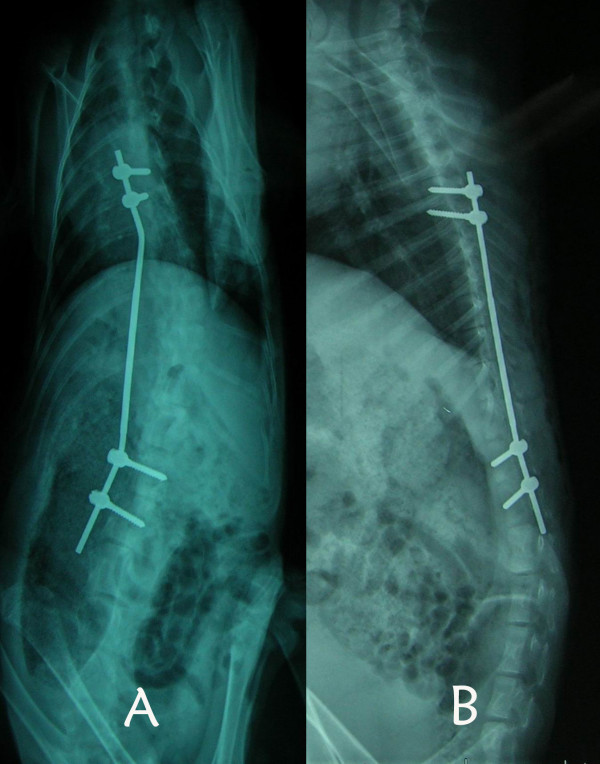
Dorsoventral and lateral plain radiographs demonstrate the unilateral pedicle asymmetric tethering in combination with contralateral rib resection. The initial Cobb angle: scoliosis 34°, kyphosis 0°, no rotation.

**Figure 4:**
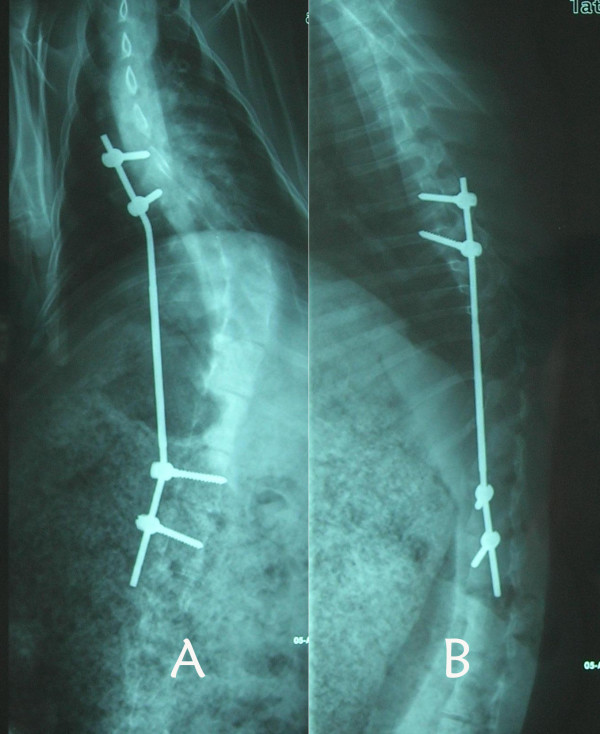
Eight weeks postoperatively (scoliosis 58°, kyphosis 10°, rotated severely).

Dorsoventral and lateral plain radiographs demonstrate scoliosis toward to the right side had been achieved after the surgery (Fig. 3). The following series radiographic examination found that 1 (8.3%) goat had nonprogressed curve and that the curvature increased with time (Fig. 4). The body weight of the nonprogressed animal did not show significant increase during the tethering period, indicating that the spines of the goats had no elongation as well (Table 1).

**Table 1:** the Cobb angles and body weights of the 12 animals

No.	Cobb angle (°)			Body weight (kg)	
	
	Pre-O	Post-O	Post-O	Pre-O	Post-O
		0 W	8 W		8 W
1	0	24	37	6.8	11.0
2	0	28	44	8.0	13.2
3	0	35	50	7.2	12.5
4	0	27	27	6.5	6.5
5	0	34	58	6.8	14.0
6	0	23	36	7.5	10.0
7	0	22	36	6.2	10.2
8	0	38	56	7.6	13.8
9	0	32	44	6.8	12.0
10	0	25	43	6.4	12.2
11	0	31	45	7.4.0	12.4
12	0	29	40	6.0	9.8

The initial scoliosis created in the progressors after the procedures measured 29.0° on average (range 23.0°–38.5°) and increased to 43.0° on average (range 36.0°–58.0°) over 8 to 10 weeks. The average progression was 14.0°. The curvature immediately after tethering surgery (the initial Cobb angle) did have a highly significant correlation with the final curvature (p < 0.001).

**Figure 5:**
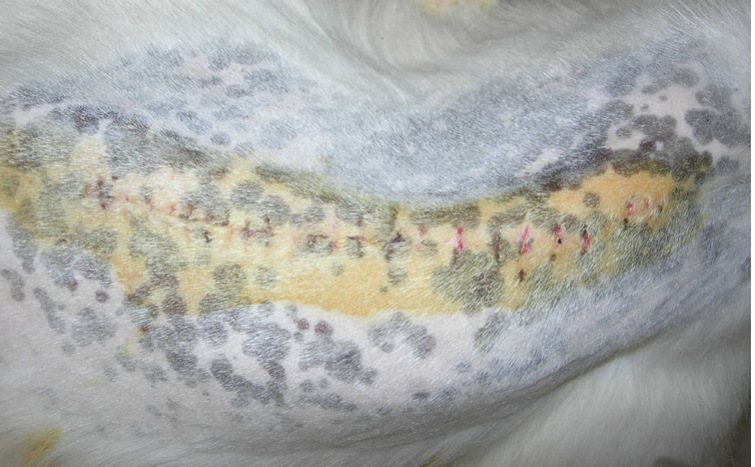
The local curvature of the thoracic spine shows a typical, posterior view of idiopathic-type scoliosis involving a right rib prominence and a left depressed thoracic cage.

Each goat with progressive curves showed a typical, posterior view idiopathic-type scoliosis with a right rib prominence and a left depressed thoracic cage (Fig. 5). The gaits of these creeper animals exhibited imbalances of the spines.

## Discussion

Normal spine growth requires a precise and delicate mechanical balance of equilibrium and postural tone. Disturbances in primary structures, supporting structures, growth centers, position of the spine, and related neural or muscular components, theoretically, could result in scoliosis in the growing animals. Therefore, by properly impacting on the balance of the spine, we can create the unique three-dimensional deformity according to our needs. Many methods had been tried to create progressive scoliotic curves, only a few successes have been achieved in large animal models. However, some shortcomings related to the current posterior asymmetric tethering were still evident.

The purpose of this study, as previously described, is to refine a minimally invasive scoliosis model in an immature goat produced by mechanically modulation of the spine, which could later be applied to human-sized technologies and devices. The ideal technique would result in a fusionless spine with a reproducible Cobb angle which would not violate the tissues surrounding the spine for future corrective treatments [26].

The development of corrective techniques for the spinal curvature in animals has paved the way for experiments on the production of such deformities. Compared to the hook techniques, pedicle screw offers less neurologic problems [27], and can be implanted or removed easily. Therefore, pedicle screw was chosen to replace the sublaminar hook as described by Braun JT.

Straightly speaking, the pedicle screw asymmetric tethering is not simply posterior tethering. It is because the compress strength has been extended to the anterolaterior vertebral body though the procedure is performed through posterior approach. There, the scoliosis is theoretically significant in this experimental model, while the lordosis is relatively less. The data of this study has also confirmed this hypothesis.

The experimental production of these curvatures are based on the recognition of four facts: 1) that the pedicle screw is strong and safe enough, 2) that epiphyseal growth can be retarded by compression [28-31], 3) that the length of the instrumental segment of the spine may increase during the tethering period, 4) that unequal elongation of the two sides of the spine will result in spinal curvature.

Asymmetric tether provides an ideal growth condition of imbalance, where the thoracic skeleton contributes a great to maintain the dynamic balance of the spine [32]. It shall be therefore taken into consideration during the mechanical modulation of the spinal growth. The elastic recover strength of the opposite side (right side) of the thoracic skeleton may reduce a lot if we the contralateral rib resected. According to the Hueter-Volkmann principle, the imbalance may increase accordingly and thus shorten the tethering period.

As the etiology of the scoliosis has not been fully understand, it is impossible to completely regenerate the special deformity. The animal model created by this method is therefore morphological rather than etiological. However, the structural alterations of these experimental models are similar to those of idiopathic deformity: scoliosis, rotation, hypokyphosis. By this mean, this method introduces a convenient way to study the correction of the deformity.

The advantages of the method to create idiopathic deformity are obvious: 1) without violating the spinal elements along the curve, 2) without extensive hemilaminotomy and sublaminar dissection, 3) easy to implant or to remove the tether relatively. 4) less anatomic limitation. The last aspect is the most significant as it is very practical in creating animal scoliotic model, i.e. it is theoretically possible to create all types of scoliotic model by implanting the pedicle screws selectively.
